# American Cyclopædia of Practical Medicine and Surgery

**Published:** 1833

**Authors:** 


ART. VIII. CYCLOPÆDIA OF PRACTICAL MEDICINE AND SURGERY.

*The Cyclopædia of Practical Medicine and Surgery, a Digest of Medical Literature.* Edited by ISAAC HAYS, M. D. Part I. Carey, Lea & Blanchard, Philadelphia, 1833.

The great *Dictionnaire des Sciences Medecales*, in 60 volumes, by the Profession in France, seems likely to be followed by many works of a similar kind, in those countries which look to that for examples. Among these are Great Britain and the United States. In the former, two cotemporary works, of more limited extent however, than that of the French, are now in the course of publication: in the latter, the work, of which the title is placed at the head of this article, is on the same plan, and may be regarded as the first attempt of the kind.

Its chieftain, Dr. Hays, is already well known, as the author of a variety of original papers, as a translator, annotator, and especially a contributor to our valuable cotemporary, the American Journal of the Medical Sciences. We have no doubt that Dr. Hays possesses many fine qualifications for the arduous and responsible office, to which he has been called. Among the names of those who are engaged as collaborators, we notice professors of no less than five medical schools, and many others who are distinguished as private lecturers or writers. This promises well. The editor informs us that—

"In the preparation of this work, free use will be made of the *Dictionnaire de Medecine et de Chirurgie Pratiques*, the *Dictionnaire de Medecine*, the *Cyclopædia of Practical Medicine*, *Copland's Dictionary of Practical Medicine*, and of the *Encyclopadisches Worterbuch der medicinischen Wissenchaften*, all now in the course of publication; and besides embracing whatever is valuable in them, it will contain a very large amount of new matter, principally relative to *American* medical practice and surgery."

On reading this candid acknowledgment, the pleasant emotions raised in us by the sight of the title-page, were somewhat abated. Our first impressions were, that an *American* Dictionary was commenced, and in its execution, would, from the names of those concerned in it, do honor to our medical literature.

We are, now, not without misgivings, that it may exhibit more naturalization than cultivation, of the medical sciences. This application of the American brand to European articles, has long been practised among us and that too, in many instances, by gentlemen whose talents fitted them for a much higher vocation. We have frequently felt disposed, to enter our humble *caveat* against this appropriation of that time and intellectual energies of our eminent teachers, and other talented physicians, which might be so much more profitably employed, in the production of original works, or at least of original compilations. We have yet to learn the advantage to the profession, of the dedications, introductions, perorations, or notes—marginal and supplementary, which envelope, like the swaddling clothes of the young infant, so many Scotch and English works, when they have their second birth on this side of the great waters; and are equally unable to comprehend or sympathise in the ambition, that prompts our distinguished physicians, to aspire to this sort of *accoucheuring*. We must, however, do our respected brethren of the present day, the justice to admit, that they are not the beginners of this custom. It commenced with the first republication of European books in this country. But can any sufficient reason be given for its continuance? In *most* cases, it certainly would be quite as well, for our publishers to reprint English, Scotch and Irish works, without having them edited. The very fact of our becoming editors and annotators, suggests the *idea* of inferiority; and if this is really the case, we are not likely to augment, in any decided manner, the value of the works upon which we direct our editorial labors, while they impress a stamp of unquestionable, if not deep degradation on our professional character.

Did we limit ourselves to the humble ambition of placing our names *beneath* those of the authors, through whose works we scatter notes, (which too often are only extracts from other books,) no objections could be raised to the practice, but that the indulgence of a harmless vanity, in certain individuals, has tended to lower the dignity of a profession, in which they enjoy a high relative rank; but the matter does not stop here. It has been, and still is, one of our employments, to turn European into American books. Nothing is easier to perform. A new title-page, a new preface, and a breaking up of the succession of chapters, by which an artificial is substituted for the natural method of the author, are the only essentials. The introduction of an occasional section or paragraph of original composition, will more fully establish the claim of the second author, to the honors which exclusively belong to the first. It is not necessary that we should designate the eminent men, who have thus spent their time in Americanizing foreign books, and appeared before the public as the authors of large octavos, which were manufactured elsewhere. Their names and their works must occur to every intelligent reader. These piracies bring discredit on the character of the profession in the United States, and it should be regarded as a duty of our periodical press, to expose them to the world.

The Cyclopædia of Practical Medicine and Surgery, upon which our attention is now directed, is intended, we presume, to be put forth as an American work; but we fear that no inconsiderable portion of it, will be a reprint of the European dictionaries enumerated in the extract we have given. These portions, it is true, may not be the least valuable part, for the physicians of Europe have more industry, and the means of wider and deeper research, than those of the United States; but still it would be agreeable to know, in reading an article, whether it was native or exotic; and we cannot, in fact, perceive any good reason why we should not reprint European Dictionaries of Medical Science, *verbatim*, until we have a profession sufficiently mature, to bring forth an *original* cyclopædia.

Situated, as we are, in the *Back woods*, we have not had the advantage of seeing the cyclopædias, which are to supply most of the *materiel* for our first dictionary of Medicine and Surgery; and are not able, therefore, to inform our readers, to what extent its contributors have wielded the scissors, instead of the quill. Among them, however, we recognise many, for whose talents and learning we cherish the highest respect; and whose labors, if directed to the production of new articles, would confer honor on the American name. It will be for them to set a good example; to aim, as much as possible, at original compositions, and indicate as borrowed, all those which are extracted from the cyclopædias, which they consult. No other course can be compatible with their character, and no other should meet the approbation of the American profession.

In reference to the first part of the work now before us, we do not mean to insinuate that this is not done; for we observe frequent references to the dictionaries and cyclopædias of Europe; but it is not always possible to find out whether the American writers have borrowed facts only, or whole pages of current composition, from those works; and, still, every intelligent and inquisitive reader, would like to know, whether that which he is studying, is foreign or domestic. He cannot divest himself of a desire to give praise or blame to him, who deserves it.

The first number consists of 108 pages, nearly four-fifths of which are devoted to the word *Abdomen*, and most of the remainder to *Abortion* and *Abscess*, the last of which is unfinished. The first article is the joint product of the editor, Dr. Hays, Dr. Reynell Coates, and Professor Geddings of Baltimore. As it presents, we may presume, a practical illustration of the plan of the work, we shall enumerate its divisions, that such of our readers, as think of subscribing, may judge for themselves.

ANATOMY OF THE ABDOMEN:—*parietes, structure of the walls, organs contained within the cavity, modifications arising from age;* —SURGICAL ANATOMY OF THE ABDOMEN;—ABNORMAL ANATOMY;—*Deviations depending on defective formative energy, depending*

*upon the opposite condition, derangement in the situation and relations of the organs.*

PHYSIOLOGY OF THE ABDOMEN;—SYMPTOMATOLOGY OF THE ABDOMEN; *temperature, form and size, tension and hardness, morbid sensations.*

PATHOLOGY OF THE ABDOMEN;—WOUNDS; *punctured, incised, contused, lacerated, gunshot;*—CONTUSIONS OF THE ABDOMEN;—RUPTURES FROM INTERNAL CAUSES;—EFFUSIONS; *of blood, of bile, of urine, of the contents of the alimentary tube, of pus;*—ABNORMAL ADHESIONS OF THE VISCERA;—FISTULÆ OF THE ABDOMEN;—FOREIGN BODIES IN THE ABDOMINAL CAVITY;—LOOSE BODIES IN THE SAME;—ABDOMINAL TUMOURS OF VARIOUS KINDS; *scirrhus, fatty;*—ENCYSTED DROPSIES;—PHLEGMONS AND ABSCESSES;—ABDOMINAL PULSATIONS.

From this analysis, the reader will perceive, that the article has a monographic character, and must, of necessity, embrace a variety of important matters. Most of them are treated with ability and copiousness; and should the future topics be handled with equal skill and learning, the work cannot fail to be highly instructive, especially to those who reside in places remote from the great libraries, from which the materials of this comprehensive work have been drawn. The division adopted in this article, is one well calculated to present a great variety of facts; and we were particularly pleased to find the clinical and the surgical anatomy of the abdomen thrown under distinct heads. This should be done for every part of the body.

As specimens of the manner in which the article is written, we shall make a few extracts, selecting chiefly those of a practical character; and, that our readers may form a judgment of the style of the three gentlemen, who have jointly composed it, we shall permit each one to be represented.

The following anatomical remarks on the normal and abnormal anatomy of the parts concerned in hernia, may serve as a specimen of the manner in which Professor Geddings has executed his portion of the task:

"The external and internal configuration of the posterior walls of the abdomen have been already described. It is divided into two lateral portions, which are symmetrical, by the perpendicular prominence formed by the projecting bodies of the lumbar vertebra. On each side of this prominence is an elongated excavation which extends from the diaphragm to Poupart's ligament; and at the latter point the posterior and anterior walls unite with each other at an acute angle. Entering into the formation of the posterior wall, we have the skin and subjacent cellular tissue, the muscles which are attached to the posterior portion of the spine, the lumbar portion of the spinal column, with its connecting ligaments and intervertebral substance, the quadratus lumbarum, psoas magnus and parvus, and iliacus internus muscles, the fascia iliac, fascia propria, peritonæum, and numerous vessels and nerves. It will be unnecessary to describe all these parts individually, as they will be considered under the appropriate heads. We shall therefore confine our observations to those parts which are situated within the cavity, and which are placed in front and by the side of the osseous structures already adverted to.

The peritonæum and fascia propria, throughout the whole extent of this region, are merely connected to the parts which are situated beneath them, by means of cellular tissue, which is exceedingly loose in its arrangement. This explains the facility with which the former membrane is drawn downwards, when any one of the abdominal organs protrudes from the cavity. The manner in which this takes place has been already explained. It is also through the loose meshes of this cellular tissue that the matter of a *lumbar* or *psoas abscess* travels, to point at the groin, thus finding an easy route behind the peritonæum, from the point of its first development, to the situation at which it manifests itself beneath Poupart's ligament. The peritonæum, in this region, does not adhere in a uniform manner to the walls of the cavity which it lines, as it does to the anterior parietes of the abdomen, but is reflected over the surface of the different organs to furnish most of them with a covering, and maintain them in their situation.

When all the organs have been removed, the region under consideration presents an inclined plane, extending from the diaphragm to Poupart's ligament, and bounded externally by the crista of the ilium; internally by the prominence occasioned by the lumbar vertebræ, and by the brim of the pelvis. This inclined plane, by uniting at Poupart's ligament with a similar surface presented by the anterior wall of the abdomen, forms an arrangement which has been compared to the expanded portion of a funnel deficient on the side towards the pelvis, the neck of which descends behind Poupart's ligament, to form an opening, or canal, which we shall presently describe under the appellation of *crural canal* or *crural ring*.

Poupart's ligament, which, as has been stated, forms the inferior termination of these two inclined planes, as it passes from the spine of the ilium to the pubis, leaves between it and the horizontal portion of the bone in an irregular elongated opening, which is partly filled up by muscles, vessels, and nerves, and partly by an arrangement of fascia, which must now be described. This opening is somewhat narrower about its middle than at its two extremities, in consequence of the pectinæal prominence of the bone, and may be compared to a figure of 8 placed horizontally. The depression situated on the outside of the prominence, is nearly filled up by the united portions of the psoas magnus and iliacus internus muscles; that on the inner side, by the crural artery and vein, and the anterior crural nerve. On the inner side of the vein, however, and between it and *Gimbernat's ligament,* there is a small annular, or rather elliptic space, which is less completely closed, and which is described under the appellation of the *crural ring.* The arrangement of these parts will be more particularly examined, after we have described the fascia which lines the posterior wall of the abdomen.

The whole extent of the iliac and lumbar regions of the abdomen are lined by a fascia, which is strong and almost aponeurotic below, but more feeble in its structure above. It was first described by Sir Astley Cooper under the appellation of *iliac fascia,* but should be considered as merely a continuation of the *fascia transversalis* already described, of which it constitutes a portion. It reposes upon the quadratus lumborum, iliacus internus, and psoas muscles, to which it is connected by loose cellular tissue. It is attached externally to the internal border of the crista of the ilium, and internally, where it reposes upon the psoas muscles, it forms a sheath for the iliac vessels, contracts an intimate adhesion with the brim of the pelvis, and descends into that cavity to constitute the pelvic fascia. (See *Pelvis.*) Inferiorly, the *iliac fascia* is arranged differently on the outer and the inner side of the *crural vessels.* On the outer side of those vessels, it passes downwards upon the anterior face of the psoas and iliac muscles, and on a level with Poupart's ligament divides into two layers, one of which is posterior and inferior, the other anterior and superior. The first continues to descend upon the face of those muscles, to become continuous with the profound, or *pectinæal* portion of the *fascia lata* of the thigh, while the second is reflected forwards, to attach itself with the whole extent of Poupart's ligament, comprised between the crural vessels and the anterior superior spinous process of the ilium, and then to become continuous with the *fascia transversalis.* In the angle formed by the separation of these two layers of fascia is lodged the *circumflex iliac artery.* (Manec, *Sur la Hernie Crurale,* p. 21, plate 2. Paris, 1826.) In consequence of this arrangement, that portion of the space behind Poupart's ligament, and between the crural vessels and the spine of the ilium, is so effectually closed up as to render a hernial protrusion in that situation impossible, or at least exceedingly rare.

At the extreme inner part of this region, the *fascia iliaca* adheres to the pubis and the superior surface of *Gimbernat's ligament.* On the outer side of this ligament, and from that point to the external limit of the crural vessels, it adheres to the *horizontal ramus* of the *pubis,* and, fortified by the expanded tendon of the psoas minor muscle, descends behind the vessels, and becomes continuous with the *profound layer* of the *fascia lata* of the groin, as already stated in reference to the external portion. It therefore forms the posterior boundary of the *crural ring,* and the posterior portion of the *sheath of the vessels,*

while the anterior is formed by the *fascia transversalis* descending in a similar manner behind Poupart's ligament, and in front of the same vessels, to become continuous with the superficial portion of the fascia lata. This disposition of the two fascia furnishes the neck, or stem, of the funnel-like arrangement to which we have adverted above, and is instrumental in the development of two parts which are highly important in relation to the subject of *crural* or *femoral hernia;* the first, the *crural canal,* consisting of the sheath of the vessels, and the second the *crural ring,* comprising the space situated between the inner part of the *crural vein* and the external margin of *Gimbernat's ligament.* (Manec *Op. Cit.*) These two parts, generally confounded with each other, should be considered apart: but to enable us to form a correct understanding of their arrangement, it will be necessary to examine first the Fascia Lata of the groin.

This is a strong fibrous aponeurosis, which invests the whole of the muscles of the thigh, and connects itself above, on the one hand, with *Poupart's ligament,* and on the other, continues with the *fascia transversalis* and *fascia iliaca,* as just described. It does not belong to the abdomen, but its intimate connection with parts which we have been considering, renders it necessary to take some notice of it in this place.

In the upper portion of the groin, and about an inch below Poupart's ligament, the fascia lata seems to be perforated by a large rounded opening for the transmission of the internal *saphena vein,* where it is about to unite with the *femoral vein.* This appearance, however, results from the division of the fascia into an internal and an external portion, which separating from each other, the first to mount upwards behind the crural vessels, the second in front of them, give rise to an opening, the inferior boundary of which presents a thick well-defined concave border, looking upwards, through which the *saphena vein,* which is superficial, dips down to discharge itself into the *femoral vein,* which is situated beneath the fascia. That portion of the *fascia lata* which is situated on the outer side of the vein, sometimes denominated superficial, or iliac portion, mounts upwards to unite itself with the whole extent of *Poupart's ligament,* from the spine of the ilium to the pubis, and to become continuous with the *fascia transversalis,* which, as has been stated above, descends behind the ligament just mentioned, to connect itself with the fascia of the groin. Its internal border, however, instead of ascending in a perpendicular direction, takes a course upwards and inwards, in front of the crural vessels, and by describing a kind of semi-circular sweep as it ascends, its internal margin forms a *crescentic* or *falciform border,* the concavity of which is directed inwards and slightly downwards, while the extremity of this border ascends behind the extreme inner part of Poupart's ligament, to blend itself with the inferior face of *Gimbernat's ligament,* which it assists in forming. This arrangement was first noticed by Hey, and it constitutes a portion of what has since been sometimes described under the appellation of *Hey's ligament.* It was denominated, by Allan Burns, *the falciform process of the fascia lata.* It forms the anterior portion of the *sheath of the*

*vessels* or the *crural canal.* The *internal, pubic,* or *pectinæal portion* of the *fascia lata* from the internal portion of the *saphena vein,* mounts upwards in front of the pectinalis muscles, and behind the crural vessels, at the same time inclining outwards, that while it gets behind the vessels, it ascends upon the anterior face of the *psoas* and *iliac muscles.* Pursuing this course, it finally reaches the level of the *horizontal ramus* of the *pubis,* with which it adheres intimately behind the vessels and on a level with the posterior boundary of the crural ring, and finally becomes continuous with the inferior and posterior layer of the *iliac fascia,* as already described. It consequently forms the posterior portion of the *crural sheath,* or *canal.*

The crural canal is that portion of the sheath of the crural vessels comprised between the point at which they engage themselves behind Poupart's ligament and the lunated opening of the fascia lata, which is traversed by the saphena vein. This canal has been confounded by Sir Astley Cooper, Cloquet, and most writers on the subject, with the *crural ring,* from which, however, it is altogether distinct, as has been very correctly represented by Manec. The crural vessels and the anterior crural nerve, as they descend from the cavity of the abdomen into the groin, are placed in the following order: the nerve is on the outer side, the artery is situated next to it, and the vein is placed on the inner side of the artery. It has been already stated, that on the outer side of these parts, the direct continuity of the *fascia iliaca* with the *fascia transversalis* completely closes up the space between Poupart's ligament and the bone, so as to render it impossible for any protrusion to take place in that situation, at any point between the artery and the spine of the ilium. Where the vessels escape from the abdomen, however, the arrangement of the parts is different: the *fascia iliaca* descends behind them to continue with the profound layer of the *fascia lata,* while the *fascia transversalis* descends in front to continue with the superficial layer of the same *fascia* and its *falciform process.* These vessels, therefore, seem to be placed between these two layers of fascia in the same manner as though they were laid between two sheets of paper. If, however, the superficial layer be slightly elevated, by pinching it in the forceps, it will be observed that several small septa, or partitions, pass backwards, from the superficial to the deep-seated layer of the fascia lata, the most exterior of which constitutes the external boundary of the nerve, the second forming a partition between the nerve and the artery, the third separating the latter vessel from the vein, while the fourth, which is the strongest of all, passes from the edge of the falciform process backwards, bounding the vein internally, and separating it at the same time from the *crural ring,* which is on its inner side. This latter portion generally reposes in contact with a mass of lymphatic glands, the vessels of which, by traversing it, give it something of a *cribriform* appearance. It constitutes the internal boundary of the *crural canal,* the external limit of which is formed by the connexion which is established between the superficial and deep-seated portions of the fascia on the outer side of the vessels and nerves. The superior opening of this canal corresponds with the point at which the vessels escape from the cavity of the abdomen, while the inferior is represented by the point at which the saphena vein perforates the fascia lata, to unite with the femoral vein. The canal, however, cannot be considered as terminating at this latter point by an open extremity, as described by Beclard and Cloquet, because the saphena vein is still contained within a sheath, which accompanies it throughout the whole extent of the thigh. The crural canal thus formed, may be considered as representing the stem, or neck, of the funnel-like arrangement above adverted to, while the expanded portion of the same is represented by the *fascia iliaca* and *fascia transversalis*, where they are spread out within the abdomen.

In consequence of the manner in which the sheath is fortified by the numerous perpendicular partitions which traverse it from before backwards, and their intimate adhesions with the vessels, it will be impossible for any protrusion to take place through it; at least such an accident, if it ever does take place, must be of very rare occurrence, though it has been conceived by Beclard and Cloquet, that an organ, escaping through this passage, might become strangulated at the point which they have denominated the *inferior opening of the crural canal.*

The *crural ring*, properly so called, is a small triangular, or rather oval opening, having its largest extremity directed outwards, situated between the crural vein and the external concave margin of *Gimbernat's ligament.* It is larger in the female than in the male, but its dimensions vary very materially even in individuals of the same sex. On an average, its transverse diameter, which is the largest, is from six to ten lines, though where the pelvis is very much developed, it may measure an inch. Internally, the *crural ring* is bounded by the sharp, crescentic border of *Gimbernat's ligament* already described; anteriorly, by *Poupart's ligament* and the tip of the *falciform process* of the *fascia lata*, which, where it is strongly developed, forms a kind of imperfect anterior wall; externally, by the *septum* which constitutes the interior boundary of the *crural canal;* and posteriorly, by the *crest* of the *pubis.* In some cases Gimbernat's ligament is less distinctly marked than usual, and in such instances the transverse diameter of the *crural ring* is always increased in the same ratio.

This opening, which constitutes the point at which the organs protrude in crural hernia, is generally partially filled up by one or more lymphatic glands, and more or less loose cellular tissue; but in addition to this, it is fortified by a kind of *fibro-membranous septum*, perforated by numerous minute orifices, which was described by Sir Astley Cooper under the appellation of *cribriform fascia*, and which Cloquet has denominated *crural septum.*

This septum, which is strong and resistant, is placed in nearly a horizontal direction, when the individual is erect, and completely closes up the crural ring, to the whole contour of which it is attached. Internally, it is intimately connected with the crescentic margin of Gimbernat's ligament, and externally, to the inner portion of the sheath of the crural vessels. Its superior face, which is directed towards the cavity of the abdomen, and which is lined by the peritonæ-

um, is slightly concave; the inferior, which is inclined downwards, is convex. Where it is strongly developed, it seems to be composed of transverse fibres, but in many cases it is altogether of a cellular structure. In all cases, it is perforated by numerous small apertures, which are traversed by the lymphatic vessels, and sometimes one or more of these small openings are occupied by a minute gland. (Cloquet, *Op. Cit.* p. 73.) When the finger is forced from the cavity of the abdomen downwards, into the crural ring, it meets with considerable resistance, occasioned by this septum; and in consequence of the manner in which it closes up the opening, it constitutes the principal obstacle to a hernial protrusion. It sometimes happens, nevertheless, that one of the small openings mentioned above becomes sufficiently dilated or ruptured to allow a portion of intestine or omentum to escape, or the septum itself is extended, and carried before the protruding organ, so as to allow the same accident to take place. (See *Hernia.*)

The most important vessel situated in the vicinity of the crural ring is the *obturator artery.* This vessel, which most usually comes off from the internal iliac, and passes from thence to the upper part of the thigh through the obturator foramen, not unfrequently departs from this arrangement, and takes its origin from the external iliac, or the crural, either separately or by a common trunk with the epigastric. This anomalous disposition was long since noticed by Haller and some of the older anatomists, and has, in modern times, been very attentively investigated, in consequence of the intimate relations of the vessel with the parts concerned in a crural hernia. Haller speaks of nine cases in which this anomalous distribution was observed. (*Icones, Anat. fasc.* 4. Nota 9.) Hesselbach met with three examples, in thirty-two bodies, in which the obturator artery was given off by the external iliac. (*Uebur den Ursprung und Verlauf der unteren Bauchdecken-Schlagader.* 1819. 1. *Abbild.*) Monro has represented the comparative frequency of the anomalous origin of the obturator artery as 1 in 20; (*Morbid Anatomy of the Stomach and Gullet*, p. 125. Edinburgh, 1830.) and this accords with the result of the experience of Velpeau. (*Anatomie Chirurgicale*, Tom. II. p. 159.) Burns observed it in 30 cases. Lawrence and Scarpa estimate the frequency of its occurrence at 1 in 10 or 12 bodies. Out of 250 subjects, one half males, the other half females, examined by Cloquet, he found the obturator artery arising from the internal iliac, on both sides, in 160, of which 87 were males and 73 females: from the epigastric, on both sides, in 56, of which 21 were males and 35 females: from the internal iliac, on one side, and the epigastric, on the other, in 28; males 15, and females 13: from the crural, in 6; males 2, females 4. (*Recherches Anat. sur les Hernies.* 4to. p. 72.) It will thus be seen, that these anomalies in the origin of the obturator artery are more frequent in the female than in the male; an observation which has also been made by Tiedemann. (*Tabulæ Arteriarum Corporis Humani.* Tab. XXX. p. 295. Carlsruhæ, 1822.) Breschet (*Memoire sur la Hernie Crurale.* Paris, 1819.) has estimated that the obturator takes its origin from the external iliac, the crural, or epigastric, in the ratio of 12 to 63; Manec, (*Recherches Anatomico-pathologiques sur la Hernie Crurale*, p. 27,) 1 in 6; and Meckel (*Manuel d'Anatomie*, Tom. II., p. 448) affirms, that if we take into account the cases in which the artery derives one branch from the internal iliac, and a second from some one of the vessels designated, the anomaly occurs almost as frequently as the natural arrangement, inasmuch as this disposition generally exists during the early months, and only becomes modified by one of these branches becoming obliterated. Consequently, that one which remains pervious will represent the obturator, whether it come from the internal or external iliac. The result of our own observations upon this point would lead us to adopt the conclusions of Cloquet, Breschet, and Manec, which are but slightly at variance with each other. Of course, we would not include the cases just adverted to, in which the vessel derives its origin from both sources.

All these anomalies are important, inasmuch as the relations of the obturator, when it takes its origin from the epigastric or the crural, are frequently so intimate with the anterior and internal portion of the protruded parts which are concerned in a crural hernia, as to expose it to the knife in operating for the relief of that disease. It has been observed, that when it comes off by a common trunk with the epigastric, and that common trunk is long; or where it arises from the crural, below Poupart's ligament, it generally twines round the internal portion of the neck of the hernial sac, and would of course be divided in dilating a stricture upwards and inwards, or directly inwards. But when the trunk of the epigastric is short, or the vessel comes off, high up, from the external iliac, it will always pass on the outer side and behind the hernial sac, and will consequently be out of the reach of the knife. This latter disposition of the artery, is more frequently observed than the former, but the cases in which the obturator artery passes in front, and on the inner side of the sac, are by no means confined to the female sex as represented by Hesselbach. Lawrence supposed that in forty cases in which the obturator artery takes its origin from the epigastric or the crural, it will not be found passing in front of the crural ring in more than one. Our own researches however, would lead us to the adoption of a different estimate, and to represent the anterior distribution of the vessel as occurring much more frequently than is here represented. Out of eight examples of the anomalous origin of the obturator contained in the museum of the University of Maryland, which we have just examined, the artery passes in front of the crural ring in three; behind, in four; and in one, it seems to pass directly across it in a transverse direction.

In addition to the vessels enumerated, the spermatic artery should also be mentioned, amongst those which are situated in the vicinity of the crural ring. It descends with the spermatic chord in an oblique direction along the course of the inguinal canal, and in the vicinity of the external abdominal ring, and is consequently situated immediately in front of the crural opening. In attempting, therefore, to dilate the stricture in crural hernia, by cutting directly upwards this vessel might be divided, as happened to Arnaud, Hey, Scarpa, and others.

The veins follow, for the most part, the course of the arteries, and are sometimes double. Occasionally, however, they depart from the natural order of their distribution, and assume new relations which it is important to note. When the obturator artery arises from the external iliac or the epigastric, it is always accompanied by its corresponding vein; but besides this, it frequently happens, even where that artery presents its natural arrangement, that there is an obturator vein opening directly into the external iliac vein, near the brim of the pelvis. This vessel, however, is always, under such circumstances, placed out of the reach of the knife, and is situated behind the hernial sac.

Manec (*Op. Cit.* p. 29. Plate 2, fig. 3, 4.) has reported a singular anomaly of the venous system of this region. A vein was observed coming off from the lower part of the external iliac vein, from whence it ascended in a tortuous direction toward the umbilicus, where it escaped from the abdomen through an opening formed by a recession of the fibres of the linea alba, and formed a loop of three or four inches in length beneath the skin. It then re-entered the cavity by the same opening, and ascended upon the left side of the umbilical ligament to the transverse fissure of the liver, where it opened into the sinus of the portal vein. A case very similar has been observed by Meniere, *Interne* of Hotel Dieu. *Archiv. Gen.* X. 381. In both these instances the situation of the anomalous vein was such, that if a crural hernia had taken place, the vessel would have occupied the internal boundary of the neck of the sac, and would of course have been divided in dilating the stricture inwards, as recommended by Gimbernat.

It may be proper in this place to advert to a pathological state of the superficial, or external epigastric vein of the abdomen, some examples of which have been observed within a few years. In a case observed by Hourmann, the history of which is reported in the *Archives Generales,* this vein was found enormously dilated in consequence of an obliteration of the inferior vena cava. (*Dictionnaire de Medecine,* Tom. I., p. 109. 2d edition.) Under these circumstances, the blood which is prevented from returning by the cava, is carried by the superficial vein in question by means of the numerous anastomoses which it forms with the branches of the axillary vein; this collateral venous circulation, together with the azygos, compensating for the obliteration of the abdominal cava. Velpeau has also reported a case of a similar kind; and Boyer mentions another, in which the vein was dilated to the size of a child's head."

The subjoined observations on *morbid sensations* in the abdomen, are from the pen of the Editor, Dr. Hays:

"Many *morbid sensations* are experienced in the abdomen, which are all confounded together under the single epithet pain; but as each variety may have a peculiar signification, the symptomatologist should carefully distinguish them. Pain in the abdomen appears to occasion more anguish than in any other part of the body; it produces a remarkable alteration in the expression of the face, the countenance indicating not only suffering, but prostration and despair.

From their seat and peculiar characters, M Dance (*Loc. Cit.*) indicates the following varieties of abdominal pain: 1st. peritonæal, 2d. gastric or cardiac, 3d. intestinal or colic, 4th. hepatic, 5th. nephritic, 6th. vesical, and 7th. uterine. *Peritonæal* pain is acute, severe, superficial, and aggravated by the slightest pressure. *Gastric* or *cardiac*, the principal seat of which is in the epigastrium, is attended, notwithstanding its numerous grades, with more anxiety, sadness, and even despair, than any other pain. The *intestinal* or *colic* are characterized by being cutting, not constant, and accompanied with a sensation in the intestines of the movement of gas or fecal matters; they are also felt in the various parts of the abdomen when they affect the small intestines, and in the course of the colon when they are seated in the large intestine; in the rectum when the pain is attended with a frequent and often ineffectual inclination to go to stool, as is observed in dysentery, it is termed tormina and tenesmus. *Hepatic* pain has a greater tendency than any other to be converted into a sensation of weight or heaviness, generally imperfectly described by the patient. *Nephritic* pain is principally seated in the renal region, but it often extends to the bladder, testicles, or groin, following the course of the ureters, and it is sometimes intensely severe, as when a calculus is in the ureter. *Vesical* pains extend over the hypogastric region and also to the perinæum, and are aggravated by the discharge of urine which frequently occasions a burning sensation. One of the peculiar characters of *uterine* pains is that they occasion, during and after parturition, the *cutting* and intermitting pains corresponding to the contractions of the uterus. Besides these pains which are for the most part attendant on inflammations, there are others which have a different signification; they are sometimes more severe, than the preceding; as, for instance, that produced by lead colic, a pain which comes on in paroxyms and is commonly lessened by pressure.

Various other morbid sensations are experienced in the abdomen, but their characters are too indeterminate for them to furnish any assistance in diagnosis; M. Dance (*loc. cit.*) excepts from these, the epigastric uneasiness experienced by chlorotic females or those affected with profuse leucorrhœa; the lumbar and hypogastric pains which come on at the approach of menstruation, and the sensation of a ball rising up through the abdomen felt by hysteric persons; to these we may add the pain in one of the sides, especially the left, immediately under the margin of the false ribs and extending to the spine of the ilium of the same side, pointed out by Dewees (*Diseases of Females*, 1st Ed. 213.) as indicating a prolapsus of the womb. Finally, we may remark that the absence of pain and the insensibility of the abdomen to pressure, offer no conclusive evidence of the non-existence of inflammation of the viscera of this cavity. Of this, numerous cases might be quoted, some of which will be hereafter adduced.

Nearly connected with the subject of this extract, is that of *abdominal pulsations*. Having in our last number published from a correspondent, the history of a fatal case of obscure chronic disease, attended throughout with great abdominal pulsation, though no lesion of any kind was discoverable after death, we are tempted to make another extract from Dr. Hays, for the purpose of presenting what he has collected and embodied, on the subject of this phenomenon:

"*Abdominal pulsations resulting from causes not as yet well determined, and regarded as dependent upon a nervous affection.*—These pulsations are for the most part perceived principally at the epigastrium, and occasion there a throbbing, and sometimes a tumefaction, calculated to lead to a belief of the existence of an aneurism of the cœliac artery. They frequently extend also along the aorta even as far as the iliacs. In persons who are not very fat, when lying on their backs, the pulse of the aorta can always be easily detected, if pressure be made a little to the left of the median line, about half-way between the navel and the scrobiculus cordis; and in certain cases this pulsation is painfully felt by the patient himself. This occurs most commonly about the middle period of life, and appears to be more frequent in females than males. It is in nervous and hypochondriac persons, individuals labouring under derangements of digestion, who are subject to hemorrhoids, and in hysterical females, often after an obstruction or suppression of the menses, that this affection has been most frequently observed. Zuliani, (*De Apoplexia*, Lips. 1790, p. 79,) Pinel, (*Nosog. Phil.*) Albers, (*Ed. Med. Surg.* III. 12.) Senac, (*Traite des Malad. du Cœur*,) De Haen, (*Heilungs Methode, uberstebzt-von Plantner*, Leip. 1782, b. 2, s. 29.) &c. describe these pulsations as a common attendant upon hypochondriasis. Dr. Albers relates the case of a man about forty years of age, severely afflicted with hypochondriasis, attended with oppression, tendency to fainting, complete sleeplessness, &c. in whom a very strong pulsation could be felt along the whole course of the aorta, and even in the left iliac. After the use of gentle purgatives, and the discharge by stool for several days of a pitchy black matter, the above symptoms ceased, and the pulsation abated, but continued perceptible for nine months afterwards. This affection is well known to be a frequent attendant upon various disorders of the digestive organs. Lewenhoeck relates a case which lasted three days, during which the digestive organs were much disordered. (*Philos. Trans.* Abr.VII. 683.) The following case is related by Dr. Albers: A young woman, whilst menstruating, and who had been for some days constipated, was seized with fainting fits and febrile symptoms, occasionally voiding from her bowels a quantity of black matter, each evacuation of which was followed by a swoon. One morning at five o'clock, Dr. Albers was sent for, as the patient was beli[illegible]ed to be dying. She was extremely exhausted, and the fainting fits succeeded each other almost without intermission. She was only able to tell, in an under voice, that she felt a palpitation in her belly; and when Dr. Albers applied his hand to the part, he felt a violent pulsation extending from the ensiform cartilage to almost the bifurcation of the aorta. "The pulsation of the *heart* was *weaker than natural*, the pulse at the wrist extremely small, and not synchronous with the pulsation in the abdomen." Dr. Albers and Mr. Meyerhoff at first believed the patient to be affected with aneurism; but Dr. Weinholt, recollecting some similar cases recorded by Morgagni, entertained a different opinion, and recommended perseverance in the employment of laxatives and clysters, combining some opium with the former. Under the use of these remedies, in a few days the pulsation in the abdomen and tightness of the chest diminished. The stools were at first of the colour of chocolate, but afterwards resumed their natural appearance. In a short time the patient got well, and remained so several years afterwards. (*Ed. Med. and Surg. Journ.* III. 8.) Thilenius, (*Med. Chir. Bemerk.* Frankf. 1789. s. 211–217,) and Mr. Hodgson, (*On the Diseases of Arteries and Veins*, p. 96,) notice these pulsations as occurring in persons affected with a flatulence of the stomach. Hau, (*Diss. de Gastrodynia*, Upsal, 1797,) has met with them in cases of gastrodynia. Professor Chapman, in his lectures, states, that he attended a female in whom the pulsations were so violent, as actually to raise up the bed-clothes. They appeared to depend upon disordered digestion, and were cured by remedies directed to that affection. Albers has met with these pulsations in cases of hemorrhage from the intestinal canal; and he says that hemorrhoidal patients, especially when inconvenienced by compression of the tumours, often complain of throbbings about the spleen, which are distinctly perceptible to the touch; and pulsations in this last situation were also experienced in a labourer who was subject to bilious attacks, and whose case is narrated by Tulpius, (*Obs. Med.* Amst. 1652. Lib. II. Chap. 28.)

Morgagni, (Epist. 39, art. 18.) describes the case of a woman forty-four years of age, who, after a suppression of the menses for some months, was attacked with palpitations in the epigastrium; and there was an obstruction of the menses, in one of the cases mentioned by Hippocrates. Dr. Albers has seen these pulsations supervene at the commencement of pregnancy and occur at every new gestation. He reports (*Loc. Cit.* p. 11.) a case in which this was so constant that the woman relied upon that sign in preference to all others. The pulsation was sometimes so violent that the husband assured Dr. A. that it might be heard distinctly. It ceased usually after the third month. Senac (*Trait. des Malad du Cœur*) has met with the affection under consideration, in chlorotic patients, and it is often present in hysterical women. Albers, (*Loc. Cit.* p. 12.) &c. Professor Mott relates (*Loc. Cit.* p. 356.) a very interesting case occurring in a female 20 years of age, of sanguine temperament, and very delicate and irritable habit; the mother of two children; who constantly since her marriage had been subject to attacks of hysteria, great difficulty of breathing, with a most frightful sense of suffocation, great palpitation of the heart, and a distressing throbbing of the arteries of the head and superior extremities. During convalescence after the birth of her second child, she was suddenly attacked with a strong pulsation in the epigastrium, opposite to the origin of the cœliac artery. This pulsation was synchronous with the action of the heart, could be seen externally, was unattended with any tumour, and was most violent in the afternoon and evening. Tonics and antispasmodics, gentle and regular exercise, and a residence in the country, improved her health, and the pulsation entirely left her.

In a case related by Teale, (*Treatise on neuralgic diseases*, Case XIII.) pulsation of the epigastrium was attendant upon spinal irritation, and we have also met with it in cases of a similar character.

All these facts seem to indicate that the pulsations under consideration are really dependent upon some nervous affection. M. Dance inclines to the belief that the solar plexus and its ramifications have something to do with their production, and he quotes, in support of this, an experiment of Sir Everard Home, instituted with the view of determining the influence of the nerves upon the arteries. Mr. H. having laid bare the carotid artery of a rabbit, applied caustic potash upon the neighbouring branches of the great sympathetic, which soon caused a violent beating of the artery. (*Trans. Roy. Soc. Lond.* 1814.) We are led, ourselves, to believe that they are, for the most part, somehow dependent upon spinal irritation, from having seen them in connexion with that affection, and from the striking similarity between the accompanying symptoms and those attendant upon that disease. A comparison of the cases to which we have just referred, with those related by Teale in his admirable work on neuralgic diseases, will bear us out in this opinion. It is not easy, however, to explain the precise mode of connexion between the cause and effect in these cases.

The *diagnosis* may generally be determined by attention to the following circumstances. These pulsations often vary in force and frequency, appear suddenly, and disappear in the same manner, and are not always synchronous with those of the heart. In aneurisms, auscultation shows an increase in the calibre of the artery; and the pulsations caused by an impulse communicated by the arteries to a tumour, arise gradually, increase in the same manner, and they are synchronous with those of the heart; and this last is also the case in aneurisms.

These pulsations being merely a secondary affection, the *treatment* must be directed to the cure of the primary disease. When this last is spinal irritation, the treatment proper for that affection, (*q. v.*) as, the local detraction of blood by cups or leeches, followed by blisters or tartar emetic ointment to the spine, are to be employed. When connected with dyspeptic symptoms, with hypochondriasis and hysteria, the treatment calculated to remove those complaints (*q. v.*) is to be resorted to. In the hypochondriacal patient whose case is related by De Haen, the pulsations were cured by active opening medicines; and in two cases related by Albers, in which there had been previous constipation, the pulsations ceased under the use, continued for some days, of mild purgatives, which evacuated a quantity of dark matter from the bowels. In the female whose case we have quoted from Morgagni, and in whom these pulsations succeeded to suppression of the menses, they were promptly cured by bleeding. Cold baths, mild tonics, and moderate exercise, will also be found useful in some cases."

The contributions of Dr. Coates are to the surgery of the abdomen; and, from their nature, constitute on the whole, the most interesting part of the monograph. The following are his observations on the treatment of wounds of the abdomen; and as the subject is one of deep importance, we shall make no apology for the length of the extract:

"These wounds may be simple, or complicated with a protrusion or with a lesion of some of the abdominal viscera. When simple, our first care should be to arrest the hemorrhage, if any divided superficial vessels are detected. The patient is then to be placed in that attitude which most completely relaxes the abdominal muscles, and the wound closed as speedily and accurately as possible. This can be generally effected by adhesive strips alone; and indeed the cases are rare which require the additional aid of the suture. Even incisions of several inches in length, complicated with protrusions, are constantly cured by these means, and when stitches have been employed after great operations on the abdomen, it has sometimes happened that hiccough, sickness, and other alarming symptoms have supervened, and could be alleviated only by removing this cause of irritation. Still it is well known that the presence of stitches passed through the peritonæum and abdominal parietes, do not always occasion material inconvenience. There are cases in which the suture must be used, and there are some epidemic states of the atmosphere, during the prevalence of which, its employment may be preferable, for reasons already mentioned. The objection urged by Mr. S. Cooper against the suture, that the stitches not unfrequently cut their way out by ulceration before they have accomplished their office, appears to us to be not a very valid one; for when no other evil consequence follows their introduction, this little additional extension of the wound is unimportant; the parts through which they make their escape generally heal behind as rapidly as they give way before them; and previous to their removal, sufficient adhesions are formed between the edges of the wound to prevent their retraction, and the purpose of the suture is in great degree accomplished. Still, the needle should never be resorted to, except under pressing emergency; and the necessity for its use will be less frequent in proportion to the manual dexterity of the surgeon in managing the adhesive strips and other dressings.

The edges of the wound being brought into accurate coaptation, the next step required is the application of a suitable bandage and compress, to give general support to the abdomen, to assist the action of the strips or suture, and to resist the tendency of the viscera to escape at the orifice. To accomplish these objects, it is generally directed that a flat compress should be applied over the simple pledget spread with cerate, which is laid upon the previous dressings. This compress is to be acted on by means of a bandage enveloping the whole abdomen.

The roller is here liable to obvious objection, as its application is difficult or impossible, without inducing improper exertion, or change of position, on the part of the patient. Nor is the many-tailed bandage, as ordinarily prepared and employed in these cases, entirely free from inconveniences. The best apparatus for producing pressure on the abdomen, is a bandage of strips, so arranged as to overlap each other in two-thirds of their width, and stitched together by a seam along the middle of their length, which should correspond with the spinal column.

When a portion of omentum or intestine protrudes through the wound, it should be returned as speedily as possible, care being exercised to handle these parts as little and as gently as may be. If, as sometimes happens in extensive incisions, the protruding parts have dirt or any foreign matter adhering to their surface, this should be carefully removed by a stream of water gently warmed, or by the light touch of a soft sponge. If the wound is narrow, the surgeon should assure himself of the perfect reduction of the viscera by passing his finger within the peritonæum, and gently making it perform there a circular movement, so that the smooth internal surface of the membrane may be felt around the whole circumference of the incision; for it is possible that a portion of intestine may be lodged between the fasciæ of the abdomen, and thus remain without the peritonæum, while it is apparently returned into the cavity. In straight incisions, it is rarely difficult to retain the viscera within the abdomen without the use of sutures; but when irregular or angular wounds take place, they are sometimes indispensable. In England and this country, the interrupted suture is generally employed; but in France, they prefer the quilled suture, and we think with reason. This suture is applied by means of two curved needles, threaded by a flat ligature, formed of several waxed threads, doubled upon themselves, so as to make a loop at one extremity. With each of these needles the abdominal parietes are punctured from within outward, at two opposite points, distant about six or eight lines from the edge of the wound. When a sufficient number of these ligatures have been passed, an assistant pushes together the divided edges, already relaxed by the position of the patient; the surgeon introduces a small cylinder of wood, linen, or waxed cloth into the loop presented by each ligature at one of its extremities toward the lower side of the wound, and after parting the threads of the other extremity of the ribbon, he ties them in a bow-knot over a similar cylinder on the opposite side of the wound. These ligatures are allowed to remain four or five days, unless some accident in the case requires their being removed sooner. (Marjolin, *Op. Cit.*) A simple ligature of waxed silk, of suitable thickness, is perhaps preferable, upon account of the small space which it occupies, and the readiness with which it can be procured at all times.

When a portion of intestine is protruded, together with a part of the omentum, it is laid down as a standing rule, that the former should be reduced before the latter, or, in general terms, that the part which protrudes last should be reduced first; and the propriety of this advice is obvious. This object is generally accomplished without much difficulty by acting upon the intestine with the two index fingers laid in the wound, either by a steady pressure exercised by both at once, or by the alternate action of each finger separately.

Difficulty may occur in our attempts at reduction, from several causes. In small wounds the protruded intestine may be so surcharged with its ordinary contents, or with air, as to make its return by simple pressure impossible; it may be strangulated, or in a state of inflammation, or it may have formed adhesions to the edges of the wound. In larger incisions, a spasmodic contraction of the muscles may diminish the cavity of the abdomen, and dilate the wound, so that the former can scarce contain the whole bulk of the viscera, and the edges of the latter cannot be properly approximated; there may also exist a general dilatation of the intestines by gas, which may render it difficult to enclose them within the parietes of the abdomen, even when free from spasm, as was observed by Dr. J. R. Barton in the case of the unfortunate girl murdered by Gross; and also in that of a patient upon whom he operated for the reduction of a strangulated inguinal hernia, several years ago. In the former instance, the bowels were returned without puncture; but in the latter, this was impossible, and Dr. B. made a valvular opening into the protruded intestine, which he effected with a bistoury. The air being evacuated, the hernia was reduced with ease. The external wound was closed by suture and compress; but on the succeeding day the gaseous secretion had again accumulated, and reproduced the protrusion. Finally, in defiance of all opposition, the intestine forced its way through the intervals between the sutures, and the patient died. No effusion had taken place in consequence of the puncture.

The embarrassments from inflammation, strangulation, and adhesion, occur also when the omentum alone protrudes. We will notice each of these causes of difficulty in succession.

When the protruded intestine is neither inflamed nor adherent, but refuses to return in consequence of the bulk of its contents, whether solid, fluid, or gaseous, it is sometimes recommended to withdraw an additional portion of intestine, in order to distribute the contents of the fold over a larger space, so that it may be more readily urged forward through the constricted orifice into the abdomen: the intestine, thus emptied of its contents, is generally returned with ease. In pursuing this plan, it should not be forgotten that an increased amount of mesentery is involved in the wound, so that if too much intestine is withdrawn, the additional bulk may increase instead of diminishing the difficulty of evacuating the protruded part. When the distension is occasioned by air, we cannot perceive the advantage of the step just recommended; for if the protruded part is already so tightly grasped as to prevent the passage of so rare a fluid, it is hardly likely that anything short of the enlargement of the wound can facilitate the reduction.

Paré occasionally punctured the inflated intestine, in such cases, with a small needle; and Rousset, Garengeot, Sharpe, and Van Sweiten, recommend the practice; but when the puncture is made with a fine needle, the mucous coat closes the aperture almost immediately, and thus destroys its usefulness. Chopart and Dessault therefore recommended the employment of a large round needle; the use of such an instrument is perhaps still more objectionable, for the orifice may still be closed by the projection of the mucous coat, while the danger of inflammation is considerably increased. Boyer, Richerand, and Marjolin, limit the puncture for the evacuation of air to cases in which the enlargement of the wound is rendered inadmissible by some peculiarity in the circumstances; and when compelled to resort to this measure, they employ a fine triangular trochar, by means of which they are sure of accomplishing their object. A very delicate instrument would be best adapted to this operation; it should be flattened like that employed in paracentesis abdominis, and should be inserted with its edge in the direction of the length of the intestine; for the experiments of M. Travers show us, that transverse wounds of these parts are more serious than those which are longitudinal. Most of those who recommend puncturing in these cases, advise that a fine ligature should be passed round the intestine and through the mesentery at the point opposite the puncture, so that by drawing the ends of this ligature out at the external wound, we may keep the little opening in the viscus in contact with its edges, after the reduction has been effected: this is done in order to lessen the danger of extravasation; but the precaution is now generally held unnecessary. Marjolin thinks that if any fear of effusion is entertained, it would be better to seize the sides of the puncture with a dissecting forceps, and apply a delicate ligature, cutting off both the extremities close to the knot, as was successfully practised by Sir A. Cooper for a perforation of an intestine in a case of hernia. (*Op. Cit.*)

La Faye, Blancard, Sabatier, S. Cooper, &c. discard the plan entirely; and although we think that the dangers of so slight an operation have been exaggerated by some, it is difficult to conceive what circumstances can require the measure in question, except when a large portion of protruded intestine becomes dilated to such a degree that the parietes cannot be closed over it. Certainly it should never be practised when the reduction can be accomplished by the enlargement of the wound. When the protruded portion of intestine is strangulated or inflamed, no time should be lost in enlarging the wound and reducing it. Venesection, both before and after the operation, is decidedly proper, when the inflammatory action is considerable. We must not hesitate to return the inflamed intestine, whatever may be the violence of the inflammation or the peculiarity of its colour, unless it is unquestionably in a state of gangrene: it may be dark red or almost black, yet if it retains its firmness, it should be returned; but if it be in a state of mortification, other modes of proceeding become necessary, which will be noticed under the heads of wounds of the intestines and artificial anus.

In enlarging a wound of the abdomen, it is generally recommended that the incision should be carried upward, as the danger of hernia is considered greater when the injury is seated near the lower part of the cavity. The instruments commonly employed are the probe-pointed curved bistoury, with or without the aid of a grooved director. While operating, the patient should be placed in the posture best calculated to relax the part, and the surgeon holding the intestine toward the lower part of the wound, with one hand uses the index finger as a guide to the bistoury or the director. If the latter can be introduced to the bottom of the wound without difficulty, the operation may often be performed at a single effort; but if this cannot be accomplished with facility, the aponeuroses, muscles, and skin may be first divided by successive retractions of the knife, the incisions being carried from within outwards, and as nearly as possible in the direction of the fibres. Care must be exercised to avoid the track of any considerable arterial branch, and that of the suspensory ligament of the liver, for reasons already mentioned.

When, in consequence of neglect or unavoidable delay, adhesions have been formed between the protruded parts and the edges of the wound, these should be divided if possible, to permit the reduction to be accomplished. Great care is required in effecting this division, for we are compelled to carry the incisions in various directions. The probe-pointed bistoury is generally considered as the best instrument in these cases; but it would be difficult to lay down very strict rules where the circumstances of the accident may vary so widely. If the adhesions are so firm that the intestine cannot be reduced, the case is still by no means desperate when the wound is large enough to prevent the danger of strangulation; for as Callisen has observed, the protruded viscus sometimes becomes covered by granulations, and a cicatrix is formed over it.

When the spasmodic action of the abdominal muscles opposes a barrier to the reduction of the protruded bowel, or prevents the closure of the wound, we must have recourse to the free abstraction of blood by venesection, and the internal exhibition of opiate remedies, until the spasm is conquered.

In very extensive wounds of the abdomen, enormous quantities of intestine may escape. One of the most singular cases of this nature is that related by Mr. Hague, surgeon at Ripon. The patient, a lad of twelve years of age, received a wound about four inches in length, on the left side of the body, commencing about two inches below the scrobiculus cordis. "The great arch of the stomach, and the whole of the intestinal canal, (duodenum excepted) contained within the abdomen," protruded. The stomach had been evacuated by vomiting. Mr. Hague proceeded to reduce first the stomach, and then the intestine, following the course of the canal. Some difficulty was experienced in preventing the exit of the reduced viscera, from the pressure of the diaphragm in breathing, that function being performed with much labour. Five sutures were employed, and supported by adhesive strips. The patient recovered. (*Ed. Med. and Surg. Journ.* V. 129.) In cases of this description, general inflation of the bowels may render reduction very difficult; but if due care is taken to contract the wound as the intestines are returned, and to preserve the edges in contact at one extremity, by the aid of an assistant, the object can be accomplished in almost every instance. As a last resort, if all other means fail, we may then puncture the bowel with the trochar mentioned above, introducing the instrument obliquely through the several coats, and allowing the gas to escape through the canula. The instrument should be so small as not to require the closure of the puncture by ligature to prevent effusion, of which the danger is fortunately less considerable than many surgeons have represented.

The intestines themselves may be implicated in the original wound; but this is not the proper place to treat of these very serious complications: they will be discussed under the head of Wounds of the Intestines. It is also unnecessary to repeat the proper consecutive treatment of wounds of the abdomen, that subject having been already enlarged upon in the preceding section on punctured wounds.

It has been stated that the omentum may be strangulated, inflamed, or adherent to the edges of the wound, when it protrudes without being followed by intestine. If no great difficulty opposes its reduction, it should be returned immediately; but if adhesions have taken place, there are two modes of proceeding recommended under different circumstances. If the protruded portion is small, and no unpleasant symptoms are present, we may cut it off to the level of the integuments, and leave the remainder to cicatrize with the wound: this is supposed to lessen the danger of subsequent hernia. When the protruded portion is larger, or when there exists a sense of dragging about the stomach, nausea, vomiting, or other symptoms of strangulation, the wound should be enlarged immediately, and the reduction accomplished. To avoid the danger of lacerating the delicate omentum, it is best to dilate the wound from the lower angle when we have occasion to use the grooved director as a guide to the bistoury.

The omentum, when protruded from the abdomen, is sometimes attacked by gangrene. This condition is recognized by the clammy softness of the mortified part, by its colour, generally gray, but sometimes dead white, by its fetid odour, and by the discharge of a greasy fluid from its cells. In other cases, when this part is on the point of becoming gangrenous, it is hard, and of a deep-red livid colour: yet it does not bleed when cut. It was formerly advised that the gangrenous omentum should be included in a ligature placed above the dead portion; that the mortified part should be cut off, and the rest returned into the abdomen, the ends of the ligature being secured externally. This dangerous plan is now discarded; its grave inconveniences have been demonstrated by an observation of Poiteau, and by the experience of Louis and Pipelet. The proper mode of treatment in such cases is as follows: if the gangrenous portion is small, we leave it to nature; if it is voluminous, we examine it in order to assure ourselves that no fold of intestine is inclosed within it. The greater part of the dead matter should then be cut off, with the scissors, in order to relieve the patient from the disagreeable odour, and the rest enveloped with a strip of linen, spread with cerate, or moistened with weakened liquid chloride of soda. The remainder of the eschar is detached in the course of a few days, and the healthy portion lying in the bottom of the wound, closes it, and contracts strong adhesions there. The wound may also be enlarged, and after having displayed the epiploon, we may remove the gangrenous portion, by cutting through the sound parts. The reduction should then be attempted, after tying, or applying tortion to the divided arteries. This last method has the advantage of preventing the dragging produced by the adhesion of the omentum to the wound; but it greatly enhances the danger of traumatic peritonitis and consecutive hernia. (Marjolin, *Op. Cit.*)

*Gun-shot wounds penetrating the abdomen*, when not complicated with similar injury to the organs contained within the cavity, seldom require any other treatment than the free use of local and general antiphlogistic measures. They are rarely followed by protrusion of the intestines; but the ball is not unfrequently lodged within the cavity or its parietes. Little aid can be afforded by the surgeon in cases of this character. The ball may subside, by its weight, toward the lower part of the abdomen, or into the pelvis; it may remain suspended among the convolutions of the intestines, surrounded by false membranes; or it may be lodged in the spine or muscles of the back or pelvis. In some cases the presence of the foreign body gives rise to no considerable inconvenience; but in other instances it excites violent inflammation, with purulent secretion into the cavity of the peritonæum; or produces a limited abscess which may be discharged either into the same cavity, or, as sometimes happens, into the alimentary canal. In the latter case, the patient may survive. M. Marjolin relates an instance in which one of these abscesses resulting from a contusion, opened at the same time into the small intestine and the bladder; a great quantity of pus, mingled with matter from the intestine, being evacuated by the urethra: this fistulous communication had continued many years when the patient consulted him.

The majority of cases in which gun-shot wounds involve the organs within the peritonæum, terminate in the death of the patients from effusion and the consequent inflammation, during the first few days after the accident. Even when this immediate danger is past, the effusion may still take place at a later period, on the separation of the sloughs: this last occurrence, however, is very rare, as it can only happen when the peritonæum has failed to form adhesions around the eschar.

The number of recoveries, notwithstanding the formidable dangers which surround these injuries, is very considerable; but for the details of cases and treatment, we must refer the reader to the articles headed *Intestines*, *Bladder*, *Stomach*, &c., where the diagnosis and surgical management of particular accidents will be discussed more fully than would be proper in this general sketch."

The article Abortion, is from the veteran pen of Professor Dewees. The following are his remarks on the use of opium:

"Opium, in some form or other, is oftentimes of the highest consequence, and should never be omitted after the system is properly prepared for it by rest, and, if necessary, by blood-letting. Much judgment is, however, required for its exhibition, that it may not do injury. It should never be given when the pulse is highly excited, or when its operation is habitually unfriendly to the system. In the first instance it should be preceded by rest and the loss of blood; in the second, the Ext. Hyosciam. may be substituted with much advantage. In such cases this remedy should be given in proper quantities to calm the system; and it must be repeated in sufficient doses, and at proper intervals, to overcome uterine contraction. It may be administered by the mouth; or by the rectum, in the form of injection, or suppository. The use, however of opium, has its limit; for it must not be persevered in after it is ascertained that it would be neither useful nor practicable, to subdue uterine contraction; and this must be ascertained by an examination per vaginam, as recommended above, and if the os uteri be found open, or the neck distended, it would be hurtful to persevere with the opium; or when the mammæ have become suddenly flaccid, after having been distended and painful, it would be found equally useless; for in this instance, the embryo, or fœtus, has certainly lost its life, and it would be mischievous to interrupt pains, as it is by their agency alone that the lifeless ovum can be expelled; and the quicker this is done, the better for the safety of the mother."

Of Astringents, he prefers the acetate of lead, extract of rhatany, and a decoction of red rose leaves, made by boiling half an ounce of the petals, in a pint of water. The treatment, both constitutional and local, which the professor recommends, is of course judicious, because the result of long and diversified experience. We may be permitted, however, to add to it, the use of large enemata of iced water, and, when the patient becomes greatly reduced, the application of sinapisms to the epigastrium.

We have, thus, given a pretty full, and, as we think, fair notice, of the first part of the Cyclopædia of Practical Medicine and Surgery, a work that cannot fail to prove acceptable to the Profession in the United States, whether it should turn out to be a new compilation from *original authors*, or a compilation from other *compilations*. We sincerely wish well to the enterprise, and shall continue to announce its progress, from time to time, laying such portions of it before our readers, as may seem to be of paramount interest.

The work is expected to be comprised in 40 parts, of 112 pages each, making eight large octavo volumes. The price of each part is fifty cents, or twenty dollars for the set.

